# The Importance of Being Non-Defective: A Mini Review Dedicated to the Memory of Jan Svoboda

**DOI:** 10.3390/v11010080

**Published:** 2019-01-18

**Authors:** Peter K. Vogt

**Affiliations:** Department of Molecular Medicine, The Scripps Research Institute, La Jolla, CA 92037, USA; pkvogt@scripps.edu; Tel.: +1-858-784-9728

**Keywords:** Prague strain of Rous sarcoma virus, oncogenesis in mammalian cells, avian leukosis and sarcoma virus, viral genetics, oncogenes, expression vectors

## Abstract

Jan Svoboda triggered investigations on non-defective avian sarcoma viruses. These viruses were a critical factor in the genetic understanding of retroviruses. They provided the single and unique access to the field and facilitated the discovery of the first oncogene *src* and of the cellular origin of retroviral oncogenes. They continue to be of importance as singularly effective expression vectors that have provided insights into the molecular functions of numerous oncogenes. Combined with the contributions to the validation of the provirus hypothesis, Jan Svoboda’s investigations of non-defective avian sarcoma viruses have shaped a large and important part of retrovirology.

At the beginning of experimental tumor virology stands a new technique: the focus assay for Rous sarcoma virus (RSV) [1]. It transformed tumor virology into a cell culture-based science. This assay provided the technical framework for a strategy adopted from phage research which included a reductionist approach and quantitation rather than description. It also led to an emphasis on work with single cells and single virus particles. These were novel and groundbreaking ideas which were quickly applied to a broad spectrum of tumor viruses, including an increasing number of cancer-causing viruses identified in mammals [2,3,4,5,6,7,8,9,10,11,12,13].

The original focus assay used the Bryan high-titer strain of RSV, a strain that had been selected because it generated high viral titers in chicken tumors from which working stocks were prepared at the time [14,15]. As the focus assay was widely adopted, especially in US labs, the Bryan high-titer strain of RSV gained almost exclusive dominance.

## 1. Defectiveness in Replication: The Bryan High-Titer Strain as the Prototype

Using the focus assay, Temin followed up on a hypothetical analogy between RSV and lysogenic phage [16]. He studied cell foci transformed by a single particle of the Bryan high-titer strain and discovered foci that did not release a detectable infectious virus [17]. Hanafusa carried out an extensive analysis of this non-producing state, and the conclusion of these studies was that the Bryan high-titer strain of RSV was defective in replication, requiring the assistance of a helper virus to generate infectious progeny [18]. The replication defectiveness of the Bryan high-titer strain also led to the broader conclusion that virus production was not required for oncogenic transformation. However, that extrapolation was soon challenged by electron-microscopic observations that showed the release of virus particles from non-producer cells and the recovery of infectious virus from non-producing cultures [19,20]. Most of the virus synthesized by non-producer cells turned out to carry a novel surface glycoprotein provided by an endogenous avian leukosis virus that acted as a helper virus. The corresponding cell surface receptor encoded by the S1 allele of the *Tvb* gene [21,22,23] was not present on the chicken “non-producer” cells, making the virus undetectable in the standard assay [24]. However, this subgroup E envelope protein readily mediated entry into cells of the Japanese quail, Coturnix coturnix japonica. The virus released from chicken “non-producer” cells is fully infectious and capable of reproducing in quail cell cultures. Scheele and Hanafusa precisely defined the genetic defect in the Bryan high-titer strain of RSV by showing that the viral genome lacks genetic information for the envelope protein. This information is provided by the helper virus [25].

The Bryan high-titer strain of RSV became the prototype for oncogene-carrying retroviruses. Virtually all of these acutely transforming viruses are replication-defective, because they have lost viral replicative information in exchange for acquiring cell-derived oncogenic sequences. 

## 2. Non-Defective Avian Sarcoma Viruses

The discovery of defectiveness in RSV was an exciting and important event in the evolution of tumor virology. However, there were other strains of avian sarcoma in circulation, notably in Europe. These included the Prague strain of RSV [26], the Carr–Zilber and Schmidt–Ruppin strains [27,28] and the independently isolated avian sarcoma virus referred to as Bratislava 77 (B77) [29]. Several of these strains were studied for their activity in mammals [27,28,30,31,32,33,34]. In contrast to the Bryan high-titer strain, they showed significant ability to induce tumors in rodents and other laboratory animals and to transform mammalian cells in culture. Jan Svoboda was a pioneer in this area. In one of his groundbreaking studies, he showed that rat cells transformed by the Prague strain of RSV failed to release infectious virus. However, co-cultivation of these cells with chicken embryo fibroblasts led to the production of infectious RSV [35,36,37]. This observation on mammalian cells provided more compelling evidence than could be derived from chicken “non-producing” cells for the general conclusion that infectious virus replication was not required for oncogenic transformation. Since no helper virus had to be added to achieve virus replication in the chicken–rat co-cultures, these experiments also suggested that the Prague strain of RSV was non-defective, capable of both replication and transformation. The non-defectiveness of the Prague strain of RSV was soon confirmed in avian cell culture [38]. RNA analysis also showed the Schmidt–Ruppin and Carr–Zilber strains of RSV and the B77 avian sarcoma virus to be non-defective [39,40].

The finding of non-defectiveness in several strains of avian sarcoma virus changed the focus of the field. Non-defective avian sarcoma viruses carry all replicative and transforming functions on the same RNA molecule, allowing the molecular identification and characterization of these functions and their interactions. Since non-defectiveness is a unique attribute of avian sarcoma viruses and has not been observed with any other oncogene-carrying retrovirus, avian sarcoma viruses provided the only key to establishing the foundation of retrovirus genetics. In contrast, replication-defective oncogenic retroviruses can be propagated only with a helper virus, as two-virus systems, greatly complicating genetic analysis.

## 3. Conditional and Non-Conditional Mutants

The first conditional mutants of a non-defective avian sarcoma virus that are temperature-sensitive for their ability to transform cells in culture were isolated by Kumao Toyoshima. These mutants carried multiple mutations in replicative and oncogenic functions [41,42]. Steven Martin then isolated a temperature-sensitive mutant that affected only oncogenic activity, delivering genetic evidence for a specific viral oncogene, later termed *src* [43]. The existence of this oncogene could also be deduced from biochemical data. During the replication of cloned non-defective avian sarcoma viruses, derivative viruses are generated that are no longer oncogenic but reproduce and are fully infectious [44]. At high-virus dilution, they can be separated from the parental sarcoma virus and grown independently in the cell culture. They are transformation-defective. The size of their genome is smaller than that of the parental sarcoma virus. They have lost the specific viral gene encoding oncogenic activity [45]. Temperature-sensitive mutants affecting oncogenicity map to the genome section that is deleted in the transformation-defective viruses [46]. Oncogenic and transformation-defective avian sarcoma viruses are isogenic pairs, except for the deletion of the *src* gene in the non-transforming derivative [44]. Such isogenic pairs provide the opportunity to generate a specific probe for *src* using subtractive hybridization with DNA transcripts of transforming and transformation-defective genomes. This *src* probe showed that homologous sequences were present in the genome of normal avian as well as mammalian cells [47]. Other retroviral oncogenes were also identified as cellular genes [48]. The cellular origin of oncogenes was a revolutionary discovery with far-reaching consequences. It showed that the genomes of normal cells encompass genes with oncogenic potential. In the ensuing years, the functional characteristics of these genes illuminated mechanisms of cellular signaling and regulation. This became the core of a genetic interpretation of human cancer [13,49,50,51].

Conditional and non-conditional mutants in replicative genes also provided a first insight into retrovirus gene function and genome structure [52,53,54,55,56]. The group of Peter Duesberg applied RNA fingerprinting to non-conditional and conditional mutants of avian sarcoma viruses and constructed the first map of a retroviral genome [57,58].

## 4. The Continuing Significance of Oncogenic Retroviruses

The discovery of the cellular origin of oncogenes demoted oncogenic retroviruses from originators to transport vehicles or insertional mutagenic activators of cellular oncogenic information [47,48,59,60]. Many tumor virologists became cell biologists and oncologists, but retroviruses retained significant importance as sources of novel oncogenes, as animal pathogens and as models for the construction of expression vectors. 

Replication-defective, acutely oncogenic retroviruses were identified repeatedly in spontaneous tumors in avian and mammalian species and led to the discovery of new oncogenes. Among the avian viruses, an early example is *myc*, first discovered in avian myelocytomatosis virus MC29 [61,62]. The oncogene *jun*, identified in the genome of avian sarcoma virus 17, showed the importance of transcriptional regulation in oncogenesis [63,64]. The catalytic isoform of PI 3-kinase drives tumor formation by avian sarcoma virus 16 [65] and plays a major role in human cancer [66]. The genomes of several acutely oncogenic mammalian retroviruses have also yielded important oncogenes, most notably *abl*, *ras*, *raf*, *fos*, and *kit* [13].

Avian retroviruses also remain potent pathogens in chickens, causing major losses in the poultry industry. A recent example is the outbreak of epidemics of hemangiomas and myelocytomas induced by subgroup J avian leukosis viruses in China. This subgroup of avian leukosis was first identified in 1991 in Britain [67,68], but the significance of this finding has emerged only recently [69,70,71] as this virus is spreading world-wide [72,73,74]. The evolution of avian leukosis viruses is continuing, and new envelope subgroups are likely to emerge [75]. Since poultry is one of the main sources of animal protein, these viruses are of serious concern for the world food supply.

The genome of the Schmidt–Ruppin strain of RSV has been re-engineered into a widely used and exceptionally efficient vector, referred to as RCAS, that can be used to express high levels of any protein in avian cells [76,77,78,79,80]. RCAS retains the unique features of non-defective avian sarcoma viruses: active replication and spreading to adjacent cells and expression of an inserted gene. The efficiency of the initial DNA transfection that often limits the utility of expression vectors does not affect outcome with RCAS, because this expression vector is a complete retrovirus that replicates and eventually will infect every avian cell in the culture. There is also a replication-defective version of RCAS, derived from the Bryan high-titer strain of RSV. It is dependent on an avian leukosis helper virus which allows easy changes in the envelope protein and thus its host range. The RCAS vector tool has been adapted in a limited way to mammalian cells that are engineered to express the avian retroviral receptor TVA. An RCAS version with a broader host range in mammalian cells uses the envelope gene of a mouse retrovirus [80], but mammalian cells do not support virus replication and spread relies solely on cell replication.

## 5. Idle Speculation

**Figure 1:**
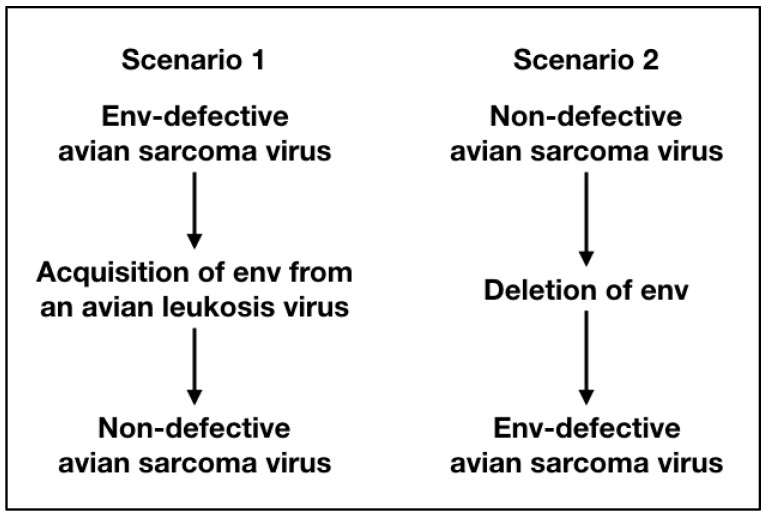
The figure summarizes the two possible genome versions of the original viral isolate of Peyton Rous and their subsequent evolution.

It has long been tempting to speculate on the origin of non-defective avian sarcoma viruses, because they are unique among acutely oncogenic, oncogene-carrying retroviruses [81]. Even among all the RSV strains, they form a separate category. The passage histories of the major RSV strains, Bryan high-titer, Schmidt–Ruppin, Carr–Zilber and Prague strains of RSV, are not known and cannot be reconstructed. Was Peyton Rous’ original isolate replication-defective or non-defective? Figure 1 summarizes these two possible scenarios.

In scenario 1, the original Rous isolate is replication-defective, lacking the envelope gene. It would have to acquire this gene to become non-defective. Although an acquisition is theoretically possible, given the high frequency of recombination between avian retroviruses, such a capture has never been observed. To detect a rare non-defective recombinant, it would be necessary to screen hundreds and possibly thousands of cell clones transformed by a single viral particle. It is unlikely for such a recombinant to become the prevalent component of an RSV stock, as defective viruses with their small genomes tend to have a selective advantage, because they replicate faster. Yet, in the non-defective avian sarcoma viruses, the non-defective component is prevalent.

In scenario 2, the *env* gene would have been deleted at some time in the passage history of certain RSV strains, including the Bryan high-titer strain. The deletions of replicative genes in avian retroviruses have been observed, so there is precedence for such a genetic change [52,82]. Combined with a selection for high virus titers, the smaller genome of a replication-defective virus could rapidly become the prevalent component during passage.

The sequence analysis of replication-defective RSV29, believed to be an early passage of the original Rous isolate, suggests that this virus is a direct precursor of the Bryan high-titer strain [83]. The conclusion that this also indicates defectiveness of the original isolate is not warranted, as deletion of the *env* gene could have occurred and could be selected for within the presumably short passage history of RSV29 [83].

These considerations favor scenario 2. It should be noted that a replication-defective version of the *src*-carrying B77 avian retrovirus has never been identified, even in the earliest passages. This virus appears to have been non-defective at the time of its isolation from a spontaneous chicken sarcoma.

The issue of the defectiveness or non-defectiveness of the original Rous isolate brings up the more important question of the acquisition of cellular oncogenes by retroviruses. There have been reports of “recovered” or “rescued” sarcoma viruses [84,85,86] that arose after infection with a transformation-defective leukosis virus. However, in all these cases, the capture of cellular *src* can be explained by homologous recombination between viral [85,86] or cellular sequences [84]. These are not de novo acquisitions as they are mediated by residual *src* sequences in the virus or retroviral sequences persisting from a previous infection adjacent to cellular *src*. The recombination between viral and cellular genomes resulting in the incorporation of an oncogene in the virus so far has occurred only in vivo. There is no experimental system that can reproducibly generate such virus–cell recombinants.

## 6. Jan Svoboda, the Catalyst

Jan Svoboda has made important, prescient contributions to the origin and validation of the provirus hypothesis [30,35,87,88,89,90,91,92]. However, he has also triggered the development that gave the field the unique tool of non-defective, acutely oncogenic retroviruses. This resulted in important insights into retroviral genetics. It provided the first biochemical evidence that a viral oncogene is not required for viral replication. It yielded a series of conditional and non-conditional mutants that illuminated viral gene functions and served as building blocks to construct a genetic map of a simple retrovirus. The non-defective avian sarcoma viruses led to the discovery of transformation-defective derivatives, and an isogenic pair of non-defective and transformation-defective viruses yielded a *src* probe that revealed the presence of *src* sequences in the cellular genome. 

**Personal note:** I first met Jan Svoboda in 1964 at the International Conference on Avian Tumor Viruses at Duke University in Durham, North Carolina. Another particularly memorable encounter was in 1981 on the occasion of a symposium held by the International Association for Comparative Research on Leukemia and Related Diseases in Los Angeles. Jan and his wife Karin visited my home in Pasadena, and we had a long discussion about the Prague spring and its armed suppression. After Prague was liberated from Soviet domination, I visited Jan several times. Our friendship was very cordial. Since I was born in Czechoslovakia and spent my childhood there, the historical rivalry between Czechs and Germans in Czechoslovakia could have divided us, but it never entered our relationship. We were aware of it, but for both of us, the common heritage was the precious bond that prevailed.
